# Causal Roles of Ventral and Dorsal Neural Systems for Automatic and Control Self-Reference Processing: A Function Lesion Mapping Study

**DOI:** 10.3390/jcm13144170

**Published:** 2024-07-16

**Authors:** Jie Sui, Pia Rotshtein, Zhuoen Lu, Magdalena Chechlacz

**Affiliations:** 1 School of Psychology, University of Aberdeen, Aberdeen AB24 3FX, UK; 2 Neuroimaging Research Unit, University of Haifa, Haifa 3498838, Israel; 3 Centre for Human Brain Health, University of Birmingham, Birmingham B15 2TT, UK; 4 School of Psychology, University of Birmingham, Birmingham B15 2TT, UK

**Keywords:** principal component analysis, voxel-based morphometry, white matter pathway, ventral neural network, dorsal control network

## Abstract

**Background**: Humans perceive and interpret the world through the lens of self-reference processes, typically facilitating enhanced performance for the task at hand. However, this research has predominantly emphasized the automatic facet of self-reference processing, overlooking how it interacts with control processes affecting everyday situations. **Methods**: We investigated this relationship between automatic and control self-reference processing in neuropsychological patients performing self-face perception tasks and the Birmingham frontal task measuring executive functions. **Results**: Principal component analysis across tasks revealed two components: one loaded on familiarity/orientation judgments reflecting automatic self-reference processing, and the other linked to the cross task and executive function indicating control processing requirements. Voxel-based morphometry and track-wise lesion-mapping analyses showed that impairments in automatic self-reference were associated with reduced grey matter in the ventromedial prefrontal cortex and right inferior temporal gyrus, and white matter damage in the right inferior fronto-occipital fasciculus. Deficits in executive control were linked to reduced grey matter in the bilateral inferior parietal lobule and left anterior insula, and white matter disconnections in the left superior longitudinal fasciculus and arcuate fasciculus. **Conclusions**: The causal evidence suggests that automatic and control facets of self-reference processes are subserved by distinct yet integrated ventral prefrontal–temporal and dorsal frontal–parietal networks, respectively.

## 1. Introduction

Humans perceive and interpret the external world through the lens of their own sense of self [1,2,3,4,5]. This self-reference ability typically manifests as a self-prioritization effect (SPE) or self-bias, a processing advantage for self-related versus non-self information [6,7,8,9,10]. The effect occurs across cognitive domains spanning from perception, attention, and memory to decision-making [11,12,13,14,15,16,17,18,19,20,21,22,23,24,25,26,27,28,29,30,31,32]. However, due to its emphasis on automatic self-reference processing [33,34,35,36,37], this research has overlooked potential interactions with control processes that affect everyday situations. Recent theoretical frameworks propose distinct yet interactive mechanisms and neural networks for self-reference processing and cognitive control [38,39,40,41,42,43,44,45,46,47,48,49,50]. Specifically, ventral temporal–prefrontal networks support automatic self-referencing, while dorsal prefrontal–parietal networks mediate attentional and executive control processes that can regulate self-biases. Although the neural manifestation of each of these core cognitive processes is well characterized, the precise relationship between them in supporting adaptive self-prioritization remains elusive. Here, we aimed to address this question by examining the roles of automatic and executive control functions in the SPE using previously reported patient data [51,52], combined with new neuropsychological assessments of control processing, to uncover latent cognitive factors underpinning the SPE. This new approach enabled the investigation of causal neural mechanisms underlying distinct components of self-prioritization processes, transcending traditional task-based analysis.

The Self Attentional Network model (SAN [38]) hypothesizes that social stimuli, such as self-related information, automatically attract attention, facilitating the SPE. It has been suggested to be mediated through the activation of ventral prefrontal–temporal networks. When self-related stimuli are task irrelevant, however, dorsal fronto-parietal networks are engaged for top-down control over this self-bias to maintain task goals. The SAN proposes that self-prioritization emerges from dynamic interactions between these ventral and dorsal control networks through key processing nodes [53,54]—the ventromedial prefrontal cortex (vmPFC) and left posterior superior temporal sulcus (LpSTS) within the ventral network, and the bilateral prefrontal cortex (DLPFC) and intraparietal sulcus (IPS) within the dorsal network. Notably, the vmPFC plays a prominent role in self-referencing, considered crucial for facilitating automatic self-prioritization as the central self-network node [55,56,57,58,59,60]. While there is some indirect evidence supporting contributions of each core network to the SAN model, direct evidence elucidating the precise relationship between them as a whole remains lacking [61,62].

The SAN model has been supported by functional neuroimaging studies in healthy participants. During a self-matching task [16], researchers reported increased activation in the vmPFC and the LpSTS in response to self-related stimuli compared to other-related stimuli [63]. These results suggest these regions as crucial nodes within the ventral self network. Specifically, the vmPFC functions as a central node for self-representation [64,65,66,67], while the LpSTS is implicated in social attention processes [68,69]. In the reverse comparison, processing other-related stimuli elicited greater activity in the DLPFC, a key region in the dorsal fronto-parietal network. This enhanced activation in the DLPFC indicates recruitment of cognitive control processes during the processing of other-related stimuli. Moreover, participants exhibited an inverse vmPFC–DLPFC relationship when matching self-related stimuli, with the SPE on behaviors (self vs. other) positively relating to activation of the vmPFC but negatively to DLPFC activation [70,71,72]. Dynamic causal modelling analysis revealed that the strength of neural couplings from the vmPFC to the LpSTS predicted the size of the SPE, suggesting dynamic interaction between the ventral (e.g., vmPFC and LpSTS) and dorsal frontoparietal (e.g., DLPFC) networks in the control of behaviors in the presence of self-related stimuli [63]. Further evidence for the regulatory role of DLPFC in self-reference processing comes from a paradigm that directly manipulates the task relevance of self-related stimuli [73]. When self-related distractors had to be ignored, left intra-parietal sulcus (a region part of the fronto-parietal network) activity increased, suggesting front-parietal control mechanisms in averting attention from social salient self-stimuli. Furthermore, focusing on self-face stimuli, an fMRI meta-analysis revealed increased responses to self- versus an other-face in ventral regions, including medial temporal, fusiform gyrus, superior temporal, inferior parietal, anterior cingulate, and inferior frontal cortices. The correlational nature of fMRI research precludes causal inferences, necessitating converging evidence from lesions.

Supporting the SAN model, a virtual-lesion study with transcranial direct current stimulation (tDCS) [74] reported diminished self-prioritization–hypos self-bias following vmPFC suppression stimulation (cathodal) (but see [70,75]). Disrupting the processes of LpSTS with a transcranial magnetic stimulation (TMS) caused reduced performances on self-related stimuli [76]. Similarly, TMS to the right posterior temporal parietal junction led to reduced egocentric self-biases [77]. These findings are further supported by neuropsychological studies in patients with acquired lesion, manifesting hypo or hyper self-biases. In a neuropsychological study, two left neuropsychological patients showed a double dissociation of the SPE. The patients performed the self-matching task and demonstrated opposing self-prioritization impairments [78]. Relative to controls, patient SC with lesions to the prefrontal cortex expanding from the vmPFC to insula and subcortical tracts showed a diminish self-prioritization effect–hypo self-bias. In contrast, patient BR with lesions to the temporal and parietal cortices, including the LpSTS, exhibited an enhanced self-prioritization–hyper self-bias. It is important to note that although the studies above demonstrated that distribution to the normal functionality of nodes within the SAN led to alteration in SPE, the direction of reported alteration was not consistent. Specifically, a virtual lesion to the left pSTS led to hypo self-bias [76], while an acquired lesion led to hyper self-bias [78]. We suggest that the left temporal–parietal region consists of multiple sub-nodes that contribute to the SAN, as revealed by the fine-tuned voxel-based analysis.

The neural substrates associated with hypo and hyper self-biases were further examined using voxel-based morphometry (VBM) in a heterogenic cohort of chronic neuropsychological patients performing a familiarity categorization task with faces [52]. Patients made familiarity judgments on their own face, a personally familiar face, and a stranger’s face presented with different head orientations. Self-prioritization was computed by contrasting responses to self versus familiar other faces. Lesions to the inferior temporal extending to the hippocampus and disconnection of the right inferior occipito-frontal (IFOF) and inferior lateral frontal (ILF) fasciculi were associated with hypo self-bias, a diminished self-prioritization relative to matched healthy controls. In contrast, lesions to the LpSTS and superior prefrontal cortex yielded hyper self-bias. Notably, the extent of self-bias correlated with patients’ executive function ability [52]. In a complementary study with the same cohort [51], researchers assessed the SPE using two different tasks with the same set of face images. In the orientation judgment task with faces as targets, participants judged the orientation of faces (left versus right). In the cross task with faces as distracters, participants judged the length of the horizontal or vertical feature of a cross. Hypo self-bias on the orientation task was predicted by lesions to the left anterior temporal pole, insula, superior parietal, and right superior frontal gyrus and disconnection of the left IFOF and ILF. For the cross task, hyper self-bias was predicted by lesions to the inferior parietal, superior temporal, and cingulate and disconnection of the cingulum. Critically, lesions to left supramarginal predicted SPE deficits irrespective of the task [51,52]. In summary, acquired lesion to ventral frontal and temporal regions is associated with hypo self-bias, while acquired lesion of the LpSTS is associated with hyper self-bias and lesion to a nearby area of the left supramarginal is associated with hypo-self.

Although the neuropsychological findings yield causal insights into the neural mechanisms of the SPE, a key challenge when making inferences from specific tasks is that the mapping of tasks to the underlying cognitive process of interest is not one-to-one. For example, the familiarity categorization task relies not only on self-reference processing but also face and picture recognition, episodic memory, and generic executive functions. Similarly, the face orientation task recruits spatial mapping, action perception, and generic executive function beyond self-reference per se. In addition, previous research only indirectly inferred involvement of executive functions and control processing. In other words, the extent to which executive function contributed to task performance and self-prioritization is unclear. Principal component analysis (PCA) [79] across tasks is one method that can overcome idiosyncratic effects of a specific self-task while directly quantifying the contribution of executive function to self-reference.

In the present study, to search for causal neural mechanisms underpinning automatic and control self-reference processing for the SPE, we combined PCA across multiple tasks with function lesion-mapping techniques. Specifically, we re-analyzed data from two previous studies assessing the SPE in different manners [51,52], combined with new neuropsychological assessments of executive functions using the Birmingham frontal task [80], to uncover latent cognitive factors underpinning the SPE. This combined approach enabled investigation of the causal neural mechanisms that underscore distinct manifestations of self-prioritization processes. Most tasks assessing self-related processing require cognitive flexibility to switch between self-related and non-self-related information. That is, processing non-self-related stimuli requires switching away from the self-prioritization rule to enable processing of other stimuli. Thus, we were particularly interested in examining the flexibility of executive functions, as measured by the Birmingham frontal task. We computed the SPE (self vs. familiar other) separately from each of the self-face processing tasks across the two studies for (i) the face familiarity categorization, (ii) a face orientation task, and (iii) the cross judgment task, where participants were instructed to disregard face stimuli while assessing the relative length of elements (horizontal versus vertical) of a cross superimposed on the faces. The Birmingham frontal task assessed patient’s flexibility in learning and switching rules to predict a dot movement on a grid. The PCA was performed on the three face SPEs and the Birmingham frontal task. We computed function lesion mapping of grey matter using VBM analysis [81], and track-wise lesion deficits [82] for white matter disconnection associated with cognitive deficits.

## 2. Materials and Methods

### 2.1. Participants

#### 2.1.1. Patients

**Figure 1:**
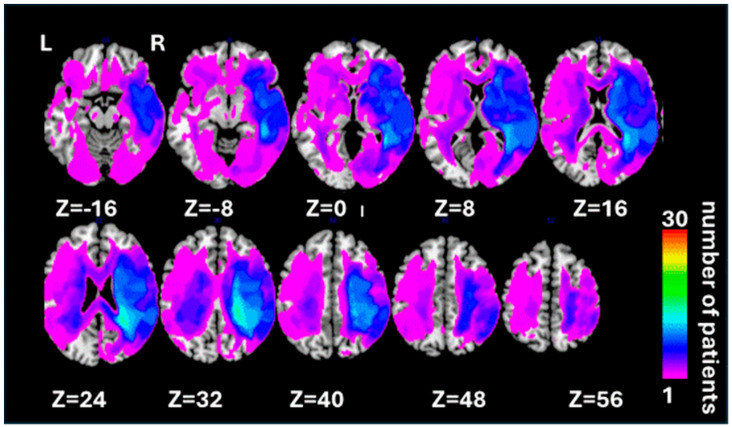
Lesion overlap map representing the spatial distribution of lesions among all 30 patients included in the current study. Lesion maps from individual patients were reconstructed based on [83]; see the Section 2—Materials and Methods for details. The lesion overlap map is shown for ten axial slices in standard MNI space with given MNI Z-coordinates of the presented axial sections. The color bar shows the number of patients with a lesion within a particular voxel (range 1–30).

We recruited 30 patients (age range from 36 to 78 years; mean 64.97 ± 10.91 years, 3 women, 27 men) from the panel of neuropsychological volunteers at the School of Psychology, University of Birmingham (Supplementary Table S1 shows demographic and clinical data for individual patients). All patients had chronic acquired brain lesions (i.e., were recruited at least 6 months post-injury). A total of 25 of 30 patients suffered stroke, 3 with carbon monoxide poisoning, 1 with herpes simplex encephalitis, and 1 with cortico-basal degeneration. All patients who participated in the study had no prosopagnosia and no contraindications to MRI. Prior to neuropsychological testing, each patient was presented with central images and required to discriminate their own faces, the faces of familiar others, and those of unfamiliar people. Participation was contingent on 100% discrimination accuracy. No other exclusion criteria were used. All patients provided written informed consent, in agreement with ethics protocols at the School of Psychology and Birmingham University Imaging Centre (BUIC). Lesion overlap across all patients is illustrated in Figure 1.

#### 2.1.2. Healthy Controls

For the lesion identification protocol (see below), we acquired T1-weighted images from 100 healthy controls (55 males and 45 females, mean age 54.5 years, range 20–87) with no history of stroke, brain damage, or neurological disorders. All the controls provided written informed consent in agreement with ethics protocols at the School of Psychology and the Birmingham University Imaging Centre (BUIC).

### 2.2. Neuropsychological Assessments

**Figure 2:**
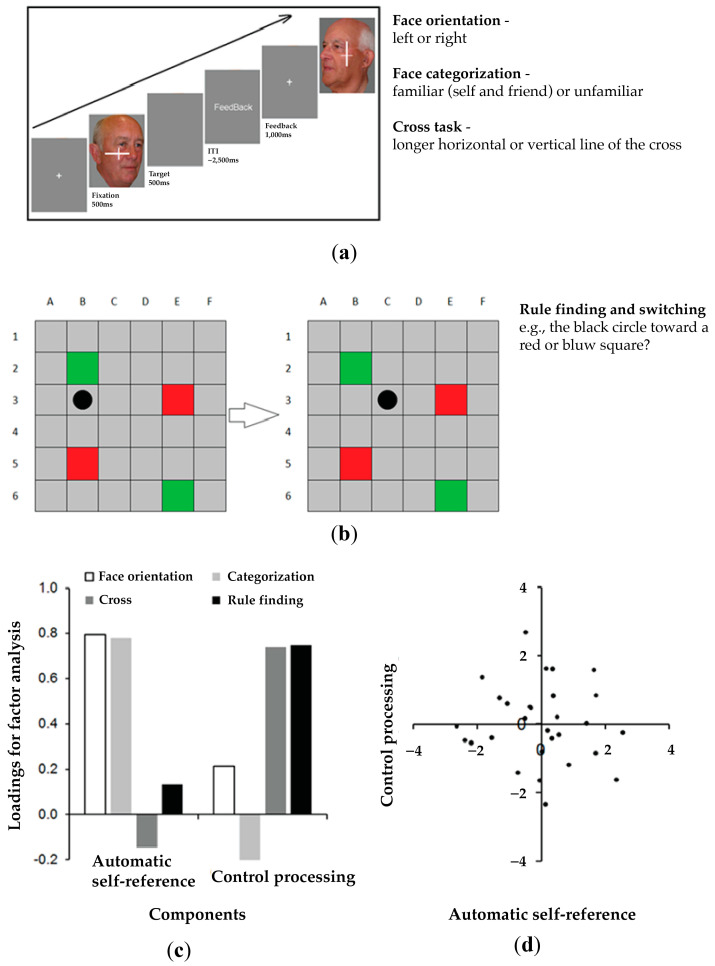
**Neuropsychological assessments.** (**a**) Experimental stimuli and protocols in the face tasks. Participants display their own face, the face of a friend, or the face of a stranger. They have to judge the orientation of the face in the face orientation task and categorize faces into the familiar (self and friend) or unfamiliar category in the categorization task. In the cross task, a cross appears on the top of the face, where participants are required to judge which horizontal or vertical element of the cross is longer while ignoring the face in the background. (**b**) In the rule-finding and -switching task in the Birmingham frontal task, participants are asked to predict the next movement of the black dot. (**c**) Principal component analysis identifies two components among the four assessments and the loadings of the four assessments for the automatic self-reference and control processing components. (**d**) No significant correlation between the two components demonstrates their separate functions of the four assessments (the distribution of participants’ loading scores in the two components).

The study employed four tasks, as illustrated in Figure 2, including a face orientation task, a cross task, a categorization task, and a rule-finding and -switching task.

In the face orientation task, participants were presented with images of their own face (self), the face of a familiar other (friend), and the face of an unfamiliar person (stranger), and they had to judge the orientation of the face but not the identity. In the cross task, there was a cross simultaneously presented with the face image and participants had to judge which horizontal or vertical element of the cross was longer while ignoring the face in the background. In the categorization task, they were asked to classify the identity of the face stimuli into one of two groups, familiar (self and familiar other/friend) or unfamiliar (unfamiliar other/stranger). We took six photographs of each participant’s face and six images of a gender-matched other person who was highly personally familiar to the participant. All images were taken with a neutral facial expression, comprising 3 left profiles and 3 right profiles of each individual. Each image was depicted at angles ranging from 15° to 45° in both directions. A gender-matched, unfamiliar other was randomly selected from the image dataset for inclusion in the current study.

In each task, the images subtended about 5° × 5° of the visual angle at a viewing distance of 60 cm. Each patient completed two blocks of seventy-two trials, with equal numbers of images in the self, familiar, and unfamiliar face conditions. Within each block, thirty-six trials were facial images oriented to the left and thirty-six oriented to the right. Therefore, there were forty-eight trials per face condition (self, familiar, or unfamiliar) for data analysis. Each trial began with the presentation of a white fixation cross at the center of screen for 500 ms. A face image was then displayed at the center of the screen until the patients made a response. The maximum duration of a face was 3000 ms, and this was followed by feedback for 1000 ms. In all three of the tasks described above, the SPE in the tasks was estimated by the differential scores in reaction times between the familiar other and the self conditions dividing by the sum of the two conditions in order to reduce individual difference. Higher or lower scores indicate the severity of the deficits—hyper or hypo self-prioritization. The data were published in two previous studies [51,52].

In the rule-finding and -switching task in the Birmingham frontal tasks from the BCoS assessment battery [80], each stimulus consists of a grid made of six columns and six lines. Most cells are grey, but two are red and two are green. Participants were asked to lean to predict the movement of a black marker across the grid. The marker moves in a lawful manner but then switches the rule by which it is operating. The switch either operates along a single dimension (e.g., moving in one direction then another) or it operates across dimensions (switch from a position rule to a color rule, where the marker jumps between squares of the same color). The rule-finding score gives measures of control function [84]. The BCoS assessment battery includes healthy control norms.

Overall, in the orientation task and categorization task, self-related information was task-relevant. In contrast, self-related stimuli were task-irrelevant in the cross task, where control processing was required to inhibit self-related distractors to keep efficient responses to targets. Likewise, in the rule-finding and -switching task, patients had to keep findings the rules while switching between the rules in order to complete the task, where again executive control processing was required. Supplementary Table S1 presents patients’ performance across the tasks. These SPE and rule-finding scores were entered into the principal component analysis (PCA).

### 2.3. Principal Component Analysis (PCA)

The PCA was performed to isolate cognitive functions underlying various symptoms associated with lesion location across the whole group of patients. This aimed at identifying independent factors across the four assessments, as well as the loading of each assessment on common factors [85,86]. The individual SPEs or rule-finding score were normalized by subtracting the averaged group mean and divided by one standard deviation. Normalized data were entered into factor analyses using a principal component approach with an orthogonal rotational (Varimax) procedure. Factors were chosen based on eigen values greater than 1. The adequacy of the correlation matrix for the factor analysis was assessed with Bartlett’s test and the Kaiser–Meyer–Olkin measure [87,88].

As predicted, there were two components—one reflecting automatic self-reference processing associated with the orientation task and the categorization task, and the other reflecting control processing based on the cross task and the Birmingham frontal task. The neuropsychological assessments were then re-calculated based on the components instead of single tasks for all individual patients. Principal component scores were measured by the sum of the associated task scores multiplying the relevant loadings. The scores were then used for the VBM analysis and tract-wise lesion deficit analysis.

### 2.4. Neuroimaging Assessment

#### 2.4.1. Image Acquirement

All patients and healthy controls were scanned at the Birmingham University Imaging Centre (BUIC) on a 3T Philips Achieva MRI system with an 8-channel phased array SENSE head coil. Patients’ scans were obtained in close proximity to the time of neuropsychological assessments. The anatomical scans were acquired using a sagittal T1-weighted sequence (sagittal orientation, TE/TR = 3.8/8.4 ms, voxel size 1 × 1 × 1 mm^3^).

#### 2.4.2. Image Pre-Processing

All T1 scans (both from 30 patients and 100 controls) were first converted and reoriented using MRICro (Chris Rorden, Georgia Tech, Atlanta, GA, USA). The pre-processing of all T1 scans was completed using SPM5 (Statistical Parametric Mapping, Welcome Department of Cognitive Neurology, London, UK). All brain scans were transformed into the standard MNI space using the unified segmentation procedure [81]. The unified segmentation procedure involves tissue classification based on the signal intensity in each voxel and on a priori knowledge of the expected location of grey matter (GM), white matter (WM), and cerebrospinal fluid (CSF) in the brain. The unified segment procedure as implemented in SPM5 has been shown to be optimal for spatial normalization of lesioned brains [89]. Furthermore, to improve tissue classification and spatial normalization of lesioned brains, a modified segmentation procedure was used (see [83] for details). The modified approach was to resolve misclassification of damaged tissue by including an additional prior for an atypical tissue class (an added “extra” class) to account for the “abnormal” voxels within lesions and thus allowing for classification of the outlier voxels. The segmented images (GM and WM maps) were smoothed with an 8 mm FWHM Gaussian filter to accommodate the assumption of random field theory used in statistical analysis [90]. The choice of intermediate smoothing of an 8 mm FWHM was previously shown to be optimal for lesion detection and further analysis of segmented images [83,91]. The pre-processed GM and WM images were used for the automated lesion reconstruction, and in the analyses to determine voxel-by-voxel relationships between grey matter damage and the self and attentional control factors.

#### 2.4.3. Lesion Reconstruction

Lesion maps from individual patients were reconstructed by using a modified segmentation procedure and an outlier detection algorithm based on fuzzy clustering [83,92,93]. This procedure identifies voxels that are different in the lesioned brain compared with a set of 100 healthy controls. The GM and WM outlier voxels are then combined into a single outlier image and thresholded to generate to a binary map of the lesion [83]. The results of lesion reconstruction were verified against the patient’s T1 scans. We used the lesion maps for all patients in the track-wise lesion-deficit analysis and to calculate lesion volumes. The lesion volumes for patients were computed using Matlab 7.14/R2012a [94].

#### 2.4.4. Voxel-Based Morphometry (VBM)

To delineate anatomical structures involved in the automatic self-reference and control components and examine contributions of grey matter changes, VBM was chosen as the analysis approach [95] since it does not require patient classification with respect to anatomical symptoms and so the analysis can include patients with a wide range of damage, including left, right, and bilateral lesions. Since our patients were not pre-selected based on clinical, anatomical, or neuropsychological criteria, VBM analysis allowed us to look for common anatomical function relationships across the whole brain, irrespective of etiology (stroke, degenerative changes) [95].

We assessed the relationship between grey matter damage and the deficits in the automatic self-reference and control functions derived from the PCA, respectively, using VBM carried out with SPM8 (Statistical Parametric Mapping, Wellcome Department of Cognitive Neurology, London, UK). We used parametric statics within the framework of the general linear model [96] and performed the analyses with the segmented GM images, with deficits associated with the two components as the main covariate of interest, while including covariates, age, gender, time since lesion, and lesion volume. We report results only showing a significant effect at the *p* < 0.05 cluster level, corrected for multiple comparisons, with the amplitude of voxels surviving at *p* < 0.005 uncorrected across the whole brain and an extent threshold of 100 mm^3^ (>50 voxels). The brain coordinates are presented in the MNI space. The anatomical location of the brain regions identified in VBM analyses is based on [97], Automated Anatomical Labeling of Activations [98], and the Woolsey Brain Atlas [99].

#### 2.4.5. Track-Wise Lesion-Deficit Analysis

**Figure 3:**
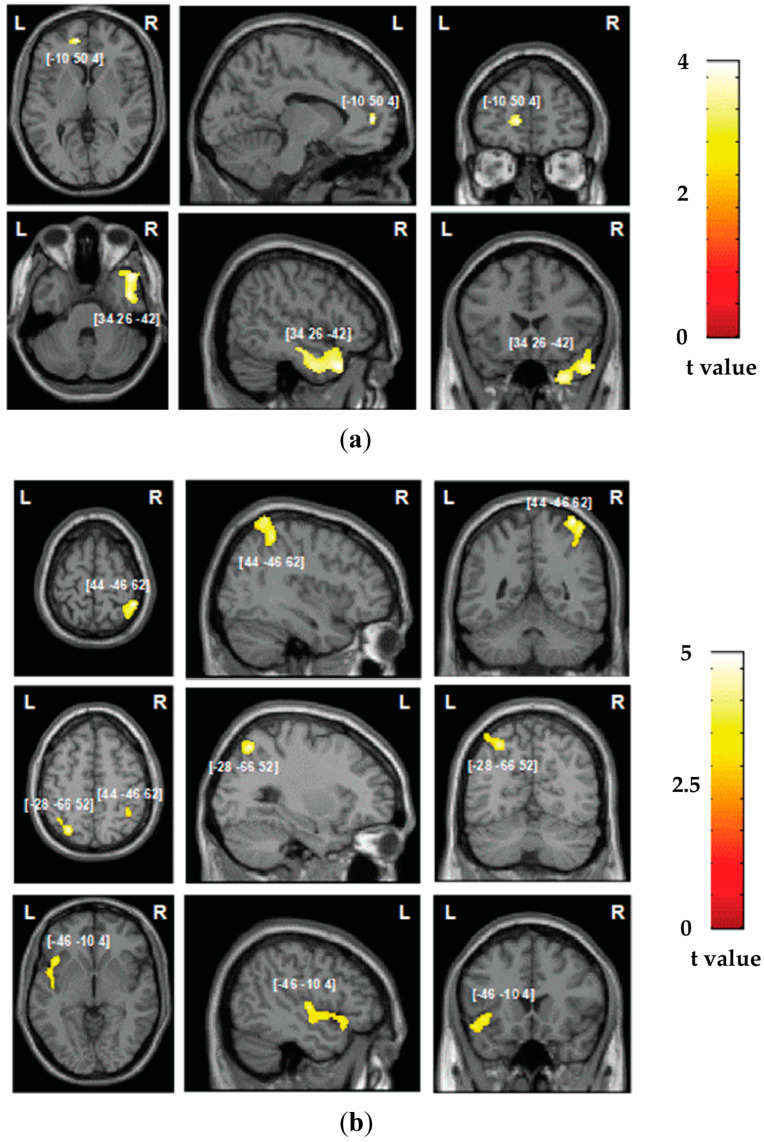
**Voxel-based morphometry analysis**: grey matter substrates of the two components identified in the principal component analysis for (**a**) automatic self-reference processing and (**b**) control processing. The areas of damage associated with both components of deficits are colored according to the level of significance in the VBM analysis, where brighter colors represent higher t-values. The numbers in brackets indicate peak MNI coordinates.

To assess the relationship between white matter damage and the deficits in the automatic self-reference and control components, we conducted tract-wise lesion-deficit analyses based on an approach [82] utilizing DTI tractography atlases of all major human white matter tracts (association, projection, and commissural) for a total of 8 pathways in each hemisphere, including the inferior frontal occipital fasciculus (IFOF), inferior longitudinal fasciculus (ILF), and uncinate, arcuate, cingulum, and 3 branches of the superior longitudinal fasciculus (SLF I, SLF II, SLFIII [100,101]). By using the patients’ reconstructed lesion maps (in MNI space), and the maps of white matter tracts from the above atlases (also in MNI space), we first evaluated the pattern of disconnection within these white matter tracts for each individual patient. All maps of white matter tracts represent a probability of a given voxel belonging to that tract, and these maps were overlapped with patients’ lesion maps. We then considered a given white matter tract to be disconnected (binary measure) if the individual patient lesion overlapped on a voxel within the white matter pathway map with a probability of at least 50% (above the chance level). Finally, we calculated the percentage of patients with the disconnection within specific white matter tracts within the left and right hemispheres, i.e., patients with versus patients without automatic self-reference processing deficits and patients with versus patients without control processing deficits (see Figure 3).

We also calculated the continuous measure of the pathway disconnection by calculating the size of the overlap (in cubic centimeters) between each patient’s lesion map and each thresholded (50%) pathway map using Matlab7.14/R2012a [94]. We used these continuous measures of white matter disconnections in the statistical track-wise lesion-deficit analyses based on linear regression. In the linear regression, we entered the lesion volume, age, and each individual pathway disconnection measure as independent variables to test whether the disconnection within specific pathways (controlling for age, injury type, and lesion location: left, right, or bilateral) predicts automatic self-reference deficits and/or control deficits (measures corresponding to the two components identified in the PCA analysis) as the dependent variable. The regression analyses were carried out separately for the left and right hemispheres. Each tract-wise lesion-deficit analysis was subjected to FDR correction. These significant effects survived FDR correction across all comparisons.

## 3. Results

### 3.1. Neuropsychological Profiles and the Self and Attentional Control Factors

The PCA yielded two components that accounted for 62% of the variance. The first component emerged from the orientation task and categorization task (automatic self-reference). The second component was associated with the cross task and rule-finding and -switching task (control processing). The two components showed a dissociation in functions between automatic self-reference and control processing. This was loaded on all variables and accounted for 62% of the variance. Factor loadings for principal component analysis using varimax rotation for the four tasks are shown in Figure 2.

### 3.2. Neuroimaging Findings: Grey Matter Damage

In the studied group of patients, the overall lesion distribution was within both hemispheres, encompassing both grey and white matter substrates and with maximum overlap within the right hemisphere, as presented in Figure 1. We subsequently assessed the neural correlates of deficits in the components of automatic self-reference and control processing identified in the PCA analysis, first employing VBM to explore the grey matter correlates of the two components. Grey matter lesions in the left ventral medial prefrontal cortex and inferior temporal gyrus were associated with the automatic self-reference component (corresponding to hypo self-bias deficits; Table 1 and Figure 3a), while grey matter lesions in the bilateral inferior parietal lobule and the left anterior insular cortex were associated with the deficits in the control component (Table 1 and Figure 3b).

### 3.3. Neuroimaging Findings: White Matter Disconnections

**Figure 4:**
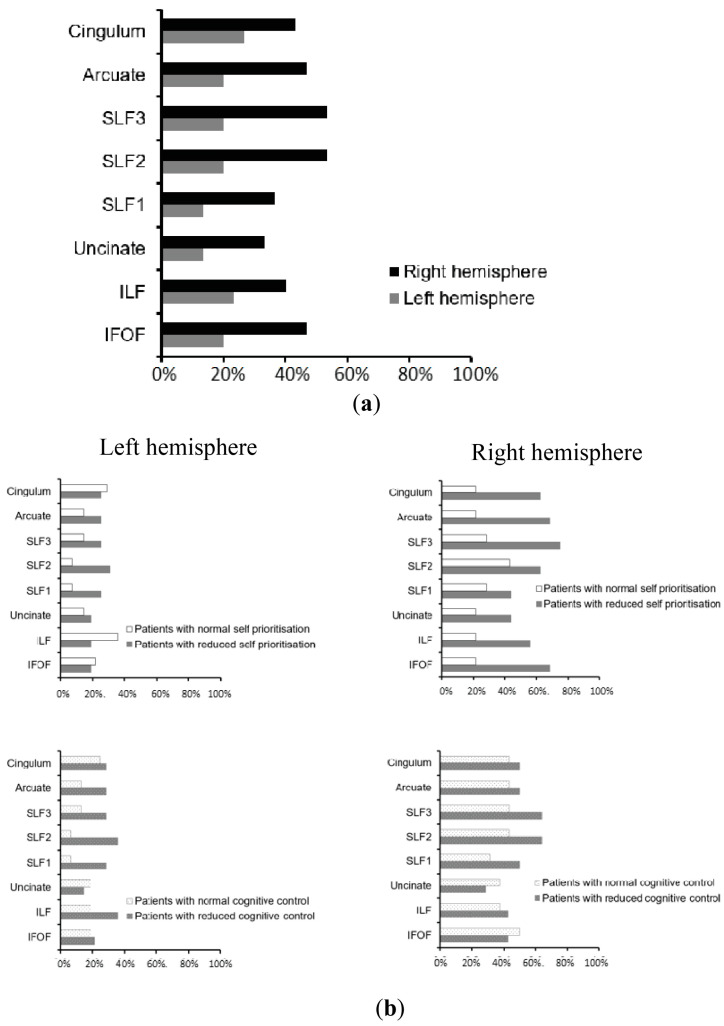
**Tract-wise lesion deficits:** (**a**). Percentage of patients with disconnection in the eight examined association, commissural, and projection white matter pathways within the left versus right hemisphere, plotted across the entire group of patients. (**b**). Percentage of patients with disconnection in the eight examined association, commissural, and projection white matter pathways within the left versus right hemisphere, calculated for groups with and without deficits in automatic self-reference and control processing (classified based on norms from healthy control participants. Note: We cannot directly classify patients with or without deficits based on the scores for the two components derived from PCA analysis). As indicated in the Methods section, the tract-wise lesion deficits include eight pathways within both the left and the right hemisphere (cingulum; arcuate; SLFI, II, III, 3 branches of the superior longitudinal fasciculus; uncinate; ILF, inferior longitudinal fasciculus; IFOF, inferior frontal occipital fasciculus).

**Figure 5:**
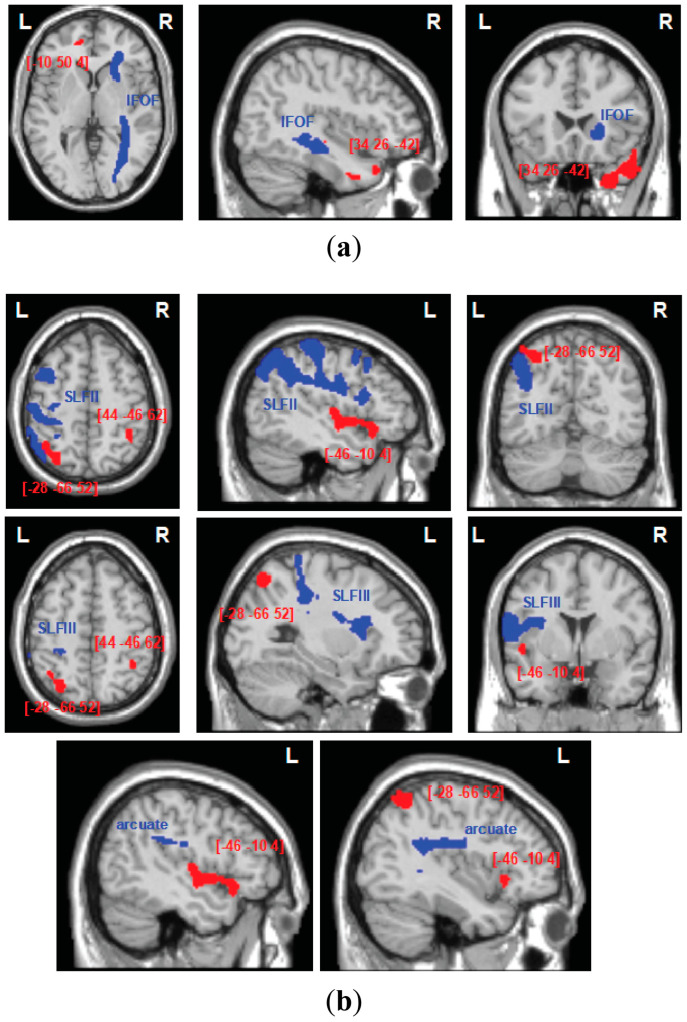
**Tract-wise lesion deficits:** the trajectories of white matter pathways (blue) presented in relation to cortical substrates for automatic self-reference (**a**) and control components (**b**), as identified in VBM analysis (red). The white matter pathways (IFOF, inferior frontal occipital fasciculus; SLF II, III, second and third branch of the superior longitudinal fasciculus; arcuate) are plotted as thresholded (50%) binary maps from the DTI tractography atlas of human white matter tracts [101,102], and the VBM results are presented as binary statistical maps thresholded at the significance level of *p* < 0.005.

The patterns of white matter disconnections in the studied group of patients are depicted in Figure 4. To identify the contribution of white matter disconnections to deficits of automatic self-reference or control processing deficits, linear regression analysis was performed for each whiter matter pathway. The analyses revealed that disconnection in the right IFOF predicted deficits in the automatic self-reference component, *F* (5, 24) = 4.018, *p* = 0.009, with an *R*^2^ of 0.456 (right IFOF: *b* = 0.452; *p* = 0.036; lesion location: *b* = 0.399; *p* = 0.031; Figure 4b, upper panel, and Figure 5a), while the left hemispheric white matter disconnections contributed specifically to the control processing deficit, *F* (5, 24) = 3.062, *p* = 0.028, with an *R*^2^ of 0.389 (left SLFII, SLFIII, and arcuate: *b* = 0.443; *p* = 0.037; lesion location: *b* = 0.413; *p* = 0.034; Figure 4b, bottom panel, and Figure 5b). The effects for other white matter pathways failed to show any reliable results after FDR correction. These regression analyses indicate that asymmetrical white matter disconnections predicted impairment in automatic self-reference and control processing (see Figure 4b and Figure 5).

## 4. Discussion

The PCA across four cognitive tasks revealed two distinct components accounting for the dynamics of the SPE, relating to automatic and control processing. This dichotomy, explaining approximately 2/3 of the variance in self-bias, elucidates the pivotal roles of automatic and controlled processes for the SPE. Neuroimaging analyses revealed the causal neural mechanisms underlying these functions—reduced grey matter in the vmPFC and right inferior temporal gyrus was associated with automatic self-reference processing deficits. This was compounded by disconnection of the right IFOF. In contrast, lesions in the bilateral dorsal parietal cortex and left inferior frontal cortex, alongside white matter damage in the left arcuate, SLF II, and SLF III fasciculus, were linked to decreased control functions. Such a dissociation between the ventral prefrontal–temporal network and the dorsal fronto-parietal control network is consistent with their roles in mediating automatic versus controlled self-reference processing.

We identified fundamental but distinct roles of the ventral prefrontal–temporal and dorsal bilaterial fronto-parietal networks in the SPE, supporting the SAN model. Our causal evaluation showed the critical functions of the vmPFC, right inferior temporal gyrus (ITG), and right IFOF in automatic self-reference processing for self-bias. These results were partially consistent with previous evidence that damage to the right inferior temporal and right IFOF disconnection reduced the SPE [52]. However, the role of vmPFC was newly revealed. These results likely stem from our cross task approach capturing this region’s broader self-reference functions. Indeed, capturing robust latent variables across multiple task contexts, enabled by our PAC approach, may be critical for fully understanding the contributions of key nodes like vmPFC and connectivity to self-reference in stroke patients typically with bilateral hemisphere lesions (Figure 1). Specifically, the vmPFC constitutes a core “ventral self” network prominently engaged in many self-reference tasks [56,57]. The function of this region in self-processing is well documented, spanning from basic matching tasks to trait evaluation [63,64,65]. Our causal lesion evidence extends these findings, suggesting that the vmPFC may serve as a fundamental driver of automatic self-reference processing, operating independently of specific task demands [103,104,105,106,107].

The role of the vmPFC and the right ITG in automatic self-reference processing may have implications for understanding alterations in self-concept in various neuropsychiatric conditions. Disfunction of the prefrontal cortex, including the vmPFC, has been identified in major depressive disorder [108,109,110]. Recent research also reported that the right ITG might serve as a potential biomarker for major depressive disorder in individuals with childhood sexual abuse [111]. The evidence underlies the potential link between disrupted self-processing and the manifestation of depressive symptoms. Liu and colleagues provided evidence for this relationship, demonstrating that alterations in the SPE, as measured by matching tasks related to self- and emotion-processing, can predict the onset of depressive episodes in pre-clinical participants [112]. Critically, these tasks recruit the vmPFC and pSTS as central nodes in modulating the SPE [63]. This predictive ability of altered SPE indicates that subtle changes in automatic self-processing may serve as early indicators for the development of depressive symptoms. On the other hand, patients with brain disorders who retained vmPFC and SPE functionality can harness these aspects of maintained automatic self-processing to enhance cognitive performance, such as attention in patients with neglect [113].

Moreover, it has been shown that the dorsal fronto-parietal network plays a key role in regulating the SPE [63,73,78,114,115]. The current study indicated that control processing is mediated by the bilateral dorsal parietal context and left inferior frontal areas, aligning with our previous findings in specific tasks where patients with lesions to the left hemisphere, including the superior parietal lobe, cingulate gyrus, and prefrontal cortex, produced a hyper self-bias [51,52]. Critically, we found that this control function among these cortical regions may be mediated through white matter pathways, including the arcuate and superior longitudinal fasciculus. The current finding provides new evidence that the dynamic integration of automatic and control self-reference processes relies on the intricate white matter architecture linking the frontal and parietal regions, extending recent functional connectivity insights into the white matter pathways [116,117,118,119].

Our findings have several implications for theoretical models of social cognition and clinical practice. First, they provide empirical validation for the SAN account for self-prioritization, which posits a dynamic interaction between ventral self and dorsal control networks [38,40]. This interaction is crucial for understanding the relationship between the neural basis of adaptive self-reference processing and control functions in everyday situations. Second, the elucidation of the roles of specific white matter pathways in modulating self-bias opens new avenues for research into the structural and functional connectivity of the brain and how disconnections of white matter pathways produce social cognitive deficits. Importantly, understanding how disruptions in white matter pathways contribute to social cognitive deficits invites further exploration to reveal the underlying mechanisms. Third, the identification of causal neural substrates associated with deficits in self-reference and control processing post-stroke or brain injury could inform targeted therapeutic interventions to rehabilitate these cognitive and social cognitive impairments, given the potential compensatory role of these functions. It has been reported that self-association approaches can be used to enhance neuropsychological patients’ memory and attention performance [113,120]. Techniques such as TMS and tDCS could be invaluable for revealing the complex neural dynamics that govern automatic and control facets of self-reference processing for neural rehabilitation [74,121].

In conclusion, our multiple-task, function lesion-mapping approach delineated the complementary ventral self and dorsal control networks mediating automatic versus regulated components of self-prioritization via their distributed grey and white matter substrates. These causal evaluations shed lights on neurobiological models of self-reference and inform therapeutic efforts for social cognitive impairments.

## Figures and Tables

**Table 1:** Grey matter substrates of automatic self-reference and control processing components as identified in the VBM analysis.

Factor	Size (Voxels)	Z-Score	Coordinates (X, Y, Z)	Brain Structure
***Automatic self-reference***				
	1384	3.44	34 26 −42	Right ITG ^1^
	50	3.41	−10 50 4	Left vmPFC ^2^
***Control processing***				
	405	3.93	44 −46 62	Right IPL ^3^
	275	3.74	−28 −66 52	Left IPL ^3^
	343	3.39	−46 −10 4	Left AIC ^4^

^1^ ITG = inferior frontal gyrus. ^2^ vmPFC = ventral medial prefrontal cortex. ^3^ IPL = inferior parietal lobule. ^4^ AIC = anterior insular cortex.

## Data Availability

The data presented in this study are available on request from the corresponding author. The data are not publicly available due to ongoing analyses for further investigation.

## Supplementary Materials

The following supporting information can be downloaded at: https://www.mdpi.com/article/10.3390/jcm13144170/s1, Table S1: Self-prioritisation scores in the face tasks and the rule finding scores in the Birmingham frontal task in patients.
