# Supplementary material for: Causal Roles of Ventral and Dorsal Neural Systems for Automatic and Control Self-Reference Processing: A Function Lesion Mapping Study
Source: J Clin Med. 2024 Jul 16;13(14):4170. doi: 10.3390/jcm13144170 (PMC11278450; doi:10.3390/jcm13144170)
Supplement: Supplementary file 1 [file jcm-13-04170-s001.zip › jcm-3057632-supplementary.pdf]

Supplementary Table S1. Self-prioritisation scores in the face tasks and the rule finding scores in the Birmingham frontal task in patients.

ID	The orientation task	The cross task	The categorization task	Birmingham frontal task
1	-2.67	1.77	-1.60	2
2	-0.52	0.59	-2.10	3
3	-0.90	1.73	-8.08	0
4	-0.31	1.71	-4.68	1
5	1.44	1.08	2.26	3
6	0.31	1.83	2.97	2
7	3.27	1.38	2.85	3
8	1.03	0.30	-2.20	2
9	0.88	0.14	-4.90	0
10	1.33	-0.26	-2.93	3
11	3.14	3.20	-4.69	--
12	-0.09	1.09	-2.65	1
13	-0.23	0.68	-2.32	0
14	1.32	2.46	-1.01	0
15	1.62	-0.07	2.39	0
16	4.30	3.86	-10.37	1
17	-1.20	1.14	-10.75	2
18	4.67	-2.79	-0.36	1
19	3.81	0.74	4.77	--
20	6.05	1.03	-5.28	0
21	-1.32	0.56	-9.56	2
22	0.80	0.12	-0.48	0
23	-0.45	1.81	2.57	0
24	1.62	0.91	-1.56	2
25	1.06	-1.35	-2.11	3
26	0.28	1.61	-7.28	1
27	0.55	-1.09	-4.93	3
28	0.43	-0.66	-1.72	0
29	2.89	2.31	3.12	1
30	-2.67	1.77	-1.60	2
